# *Atractylodes lancea* for cholangiocarcinoma: Modulatory effects on CYP1A2 and CYP3A1 and pharmacokinetics in rats and biodistribution in mice

**DOI:** 10.1371/journal.pone.0277614

**Published:** 2022-11-14

**Authors:** Nadda Muhamad, Tullayakorn Plengsuriyakarn, Kesara Na-Bangchang

**Affiliations:** 1 Graduate Program in Bioclinical Sciences, Chulabhorn International College of Medicine, Thammasat University (Rangsit Campus), Pathumthani, Thailand; 2 Center of Excellence in Pharmacology and Molecular Biology of Malaria and Cholangiocarcinoma, Thammasat University (Rangsit Campus), Pathumthani, Thailand; 3 Drug Discovery and Development Center, Office of Advanced Science and Technology, Thammasat University (Rangsit Campus), Pathumthani, Thailand; Universidade Federal do Rio de Janeiro, BRAZIL

## Abstract

*Atractylodes lancea* (Thunb.) DC. (*A*. *lancea*: AL) is a promising candidate for the treatment of cholangiocarcinoma (bile duct cancer). The study investigated (i) the propensity of capsule formulation of the standardized extract of AL (formulated AL) to modulate mRNA and protein expression and activities of CYP1A2 and CYP3A1 in rats after long- and short-term exposure, (ii) the pharmacokinetics of atractylodin (ATD: active constituent) after long-term administration of formulated AL, and (iii) the biodistribution of atractylodin-loaded polylactic-co-glycolic acid (ATD-PLGA-NPs) in mice. To investigate CYP1A2 and CYP3A1 modulatory activities following long-term exposure, rats of both genders received oral doses of the formulated AL at 1,000 (low dose), 3,000 (medium dose), and 5,000 (high dose) mg/kg body weight daily for 12 months. For short-term effects, male rats were orally administered the formulated AL at the dose of 5,000 mg/kg body weight daily for 1, 7, 14 and 21 days. The pharmacokinetic study was conducted in male rats after administration of the formulated AL at the dose of 5,000 mg/kg body weight daily for 9 months. The biodistribution study was conducted in a male mouse receiving ATD-PLGA-NPs at the equivalent dose to ATD of 100 mg/kg body weight. The high dose of formulated AL produced an inducing effect on CYP1A2 but an inhibitory effect on CYP3A1 activities in male rats. The low dose, however, did not inhibit or induce the activities of both enzymes in male and female rats. ATD reached maximum plasma concentration (C_max_) of 359.73 ng/mL at 3 h (t_max_). Mean residence time (MRT) and terminal phase elimination half-life (t_1/2z_) were 3.03 and 0.56 h, respectively. The extent of biodistribution of ATD in mouse livers receiving ATD-PLGA-NPs was 5-fold of that receiving free ATD. Clinical use of low-dose AL should be considered to avoid potential herb-drug interactions after long-term use. ATD-PLGA-NPs is a potential drug delivery system for cholangiocarcinoma treatment.

## Introduction

Herbal medicine is increasingly being used in clinical practice worldwide for various indications [1–3]. Herbal products have been considered safe as they have been used for a long time in the past decades. Due to their complex chemical constituents, however, potential adverse interactions are the major concern when they are concomitantly administered with other herbal or modern medicines. The cytochrome P450s (CYP450s) are mixed-function oxidases that play a crucial role in the biotransformation of xeno- and endobiotics, as well as drug-drug and herb-drug interactions [4]. Although human CYP450s are present in various organs, most are expressed in the liver, with the highest expressed isoforms being CYP3A4, followed by CYP2C9/2C19, CYP1A2, and CYP2E1 [5, 6]. The most liable CYP450 isoform for the metabolism of currently used drugs is CYP3A4 (50%), followed by CYP2D6 (30%), CYP2C9/2C19 (11%), and CYP1A2 (4%) [6]. Inhibition of CYP450 metabolizing enzymes result in increased plasma/blood concentrations of the co-administered drugs and the risk of toxicity, particularly for narrow therapeutic index drugs [4]. Induction of CYP450s, on the other hand, leads to decreased plasma/blood concentrations of the co-administered drugs, leading to the loss of their pharmacological efficacy [4].

*Atractylodes lancea* (Thunb.) DC. (*A*. *lancea*: AL) is a promising candidate for the treatment of cholangiocarcinoma (bile duct cancer). It has been used in traditional Chinese medicine for rheumatic diseases, digestive disorders, night blindness, and influenza [7]. Results from our previous studies, both *in vitro* and *in vivo*, confirm the potential anti-cholangiocarcinoma activity and safety profile of dried rhizomes extract and capsule formulation of the standardized AL extract (formulated AL) [8]. The IC_50_ (half-maximal inhibitory concentration) values of the crude and formulated AL in human cholangiocarcinoma cell lines range from 23.24 ± 0.35 to 69.19 ± 3.85 μg/mL (mean ± SD) [9–11]. The anti-cholangiocarcinoma activity of AL extract (daily oral dose levels of 1,000, 3,000 and 5,000 mg/kg body weight) has also been confirmed in animal models, both in the xenograft mice and *Opisthorchis viverrini*/dimethylnitrosamine-induced cholangiocarcinoma in hamsters [12, 13]. Furthermore, the potential inhibitory effect of AL on CYP450 activities was investigated *in vitro* [14]. The extract potently inhibited CYP1A2 (IC_50_ 0.36 ± 0.02 μg/mL). The inhibitory potency for CYP2C19 was moderate (IC_50_ 16.48 ± 1.04 μg/mL), while the inhibitory potencies for CYP2D6 (IC_50_ 313.51± 51.90 μg/mL) and CYP3A4 (IC_50_ 54.36±5.64 μg/mL) were low [14]. Phase I clinical trial to evaluate the safety and pharmacokinetics of the formulated AL has been completed [15]. Results suggest a satisfactory safety profile when given orally at a single or daily dose of 1,000 mg [15]. Atractylodin (ATD: active compound) was well absorbed, reaching maximum concentration (t_max_) within 1 hour. The elimination half-life (t_1/2_) was short (1.23 hours) [15]. Phase II clinical trial in patients with advanced stage cholangiocarcinoma is underway. As long-term administration of AL is required for the treatment of cholangiocarcinoma, assessments of potential drug interactions and pharmacokinetics are essential. The current study aimed to investigate modulatory effects of the formulated AL on CYP450, and the pharmacokinetics of atractylodin (ATD) in animals. ATD is the active constituent of AL which has also been reported for anti-proliferative activity against cholangiocarcinoma cell lines [16]. The propensity of the AL formulation to modulate mRNA and protein expression and activities of CYP1A2 and CYP3A1 enzymes was investigated in rats following long- and short-term administration of AL. The pharmacokinetics of atractylodin was investigated in rats following daily doses of AL formulation for 9 months. Due to the poor solubility of ATD which might affect the bioavailability of the compound, the bioavailability and biodistribution of the polylactic-co-glycolic acid nanoparticles of ATD (ATD-PLGA-NPs) were investigated in mouse liver using matrix-assisted laser desorption ionization-imaging mass spectrometer (MALDI-IMS). The ATD-PLGA-NPs was previously developed in our laboratory with the aim of improving the solubility, and thus *in vivo* bioavailability and biodistribution of ATD [16]. The antiproliferative activity of ATD-PLGA-NPs against cholangiocarcinoma cell lines was shown to be relatively potent with an IC_50_ ranging from 16 to 23 μg/mL and selective distribution to liver [16].

## Materials and methods

### Chemicals

Capsule formulation of the standardized *A*. *lancea* extract (formulated AL) and placebo were provided by Khaolaor Laboratories Co. Ltd. (Samut Prakarn, Thailand: lot number 200121/3 and capsule number 00). Atractylodin (ATD) was obtained from WAKO (Osaka, Japan). Caffeine, phenacetin, paracetamol, PLGA (Resomer^®^ RG502, MW 12,000, inherent viscosity 0.24 dL/g), and α-cyano-4-hydroxycinnamic acid were obtained from Sigma-Aldrich (St. Louis, MO, USA). Kolliphor^®^ P 407 (poloxamer 407) was kindly provided by BASF Thailand. β-nicotinamide adenine dinucleotide phosphate tetrasodium salt-reduced form (NADPH) and nifedipine were purchased from Tokyo Chemical Industry Co. Ltd. (Tokyo, Japan). Dehydronifedipine was obtained from Toronto Research Chemical (Toronto, ON, Canada). Diazepam was purchased from Government Pharmaceutical Organization (Bangkok, Thailand). iTaq^TM^ Universal SYBR^®^ Green Supermix was purchased from Bio-Rad Laboratories, Inc. (Hercules, CA, USA). Rabbit Anti-Rat CYP1A2 and CYP3A1 polyclonal antibodies were purchased from ABclonal (MA, USA) and Merck (Darmstadt, Germany), respectively. Rabbit Anti-Rat GAPDH antibody and Goat Anti-Rabbit IgG AP-linked were purchased from Cell Signaling Technology (MA, USA). The BCIP/NBT solution AP-chromogen was obtained from Amresco (Solon, OH, USA). Methanol HPLC grade was purchased from Fisher Scientific (MA, USA).

### Animals

Male and female Wistar (WT) rats and male Sprague-Dawley (SD) rats (5–7 weeks of age, 200±20 g of body weight) were, respectively, used for the investigation of long-term and short-term modulatory effects of AL on CYP1A2 and CYP3A1 expression and activities. Male WT rats (5–7 weeks of age, 200±20 g of body weight) were used for pharmacokinetic study. A male mouse (9 weeks, 35–40 g of body weight) was used for the biodistribution study of ATD-PLGA-NPs. All animals were obtained from Nomura Siam International Co. Ltd. and were housed at the Laboratory Animal Center, Thammasat University, in environmentally controlled room temperature (22±2°C) with a 12 h light-dark cycle and 30–70% humidity. The experiments were conducted following the approved protocol from the Ethics Committee for Animal Research, Thammasat University, Thailand (No. 019/2560 for rats and No. 016/2020 for mice) and in accordance to the Guideline for the Care and Use of Laboratory Animals [17].

### Modulatory effects of formulated AL on CYP1A2 and CYP3A1 expression and activities

#### Experimental design and treatments

CYP1A2 and CYP3A1 modulatory effects of the formulated AL were investigated in WT rats following long- and short-term exposure (*Experiment I* and *Experiment II*, respectively).

*Experiment I (long-term exposure)*. The investigation of long-term modulatory effects of the formulated AL on *CYP1A2* and *CYP3A1* expression and activities was a part of the chronic toxicity testing [18]. Rats were assigned to four groups (3 males and 3 females each) to receive treatment with the formulated AL *via* intragastric gavage once daily for 12 months as follows: group 1 (low dose) 1,000 mg/kg body weight; group 2 (medium dose) 3,000 mg/kg body weight; group 3 (high dose) 5,000 mg/kg body weight; and group 4 (control) distilled water.

*Experiment II (short-term exposure)*. For the investigation of the short-term effect of the formulated AL on *CYP1A2* and *CYP3A1* expression and activities, male SD rats were assigned to four experimental groups (3 rats each) to receive treatment with formulated AL at daily doses of 5,000 mg/kg body weight *via* intragastric gavage for 1 (group 1), 7 (group 2), 14 (group 3), and 21 (group 4) days. The corresponding controlled groups (3 rats each) received treatment with placebo.

#### Effects of formulated AL on CYP1A2 and CYP3A1 mRNA expression

Total mRNA was prepared from rats’ liver according to the manufacturer’s instruction (S1 Appendix). CYP1A2 and CYP3A1 mRNA expression levels in rat liver tissues isolated from both experiments were determined using reverse transcriptase-polymerase chain reaction (RT-PCR). Specific forward and reverse primers of the target and housekeeping genes used in the experiment are presented in Table 1. iTaq^TM^ Universal SYBR^®^ Green Supermix was used for RT-PCR analysis using CFX Connect^TM^ Real-Time System (BioRad Laboratories Inc., Hercules, CA, USA). Ten microliters of the experimental mixture contained 100 ng/μL of cDNA, iTaq^TM^ Universal SYBR^®^ Green Supermix, 10 μM of each specific forward and reversed primer and water. RT-PCR cycles for *CYP1A2*, *CYP3A1* and *β-actin* were initiated by heating the reaction mixture at 95°C for 5 min for denaturation, 50 cycles at 95°C for 10 sec, and at 60°C for 10 sec. Each reaction was carried out in three independent experiments (duplicate each). The 2^-ΔΔCt^ method was applied for the calculation of the fold-change of *CYP1A2* or *CYP3A1* gene expression level of treatment compared with control. The *CYP1A2* and *CYP3A1* target gene expression was normalized to the *β-actin* housekeeping gene as follows:

ΔΔCt=[Ct(treatmentforCYP1A2orCYP3A1)−Ct(treatmentforβ−actin)]−[Ct(controlforCYP1A2orCYP3A1)−Ct(controlforβ−actin)] (1)

$${Fold\;change=2}^{-\Delta \Delta Ct}$$ (2)

**Table 1:** Specific forward and reverse primers for the analysis of CYP1A2 and CYP3A1 mRNA expression.

Gene	Primer sequence
***CYP1A2***	Forward 5’-CTGCAGAAAACAGTCCAGGA-3’
	Reverse 5’-GAGGGATGAGACCACCGTTG-3’
***CYP3A1***	Forward 5’-CAGCAGCACACTTTCCTTTGTC-3’
	Reverse 5’-CTCCTCCTGCAGTTTCTTCTGTGTA-3’
***β-actin***	Forward 5’- AACCCTAAGGCCAACCGTGAAAAG-3’
	Reverse 5’- CGACCAGAGGCATACAGGGACAAC-3’

#### Effects of formulated AL on CYP1A2 and CYP3A1 protein expression

Liver microsomal proteins was prepared according to the previously described method (S2 Appendix) [19]. The expression levels of CYP1A2 and CYP3A1 proteins in rat liver tissues were examined by Western immunoblots analysis. The prepared microsomal proteins were denatured at 100°C for 5 min, separated onto 12.5% sodium dodecyl sulfate-polyacrylamide gel electrophoresis (SDS-PAGE) (25 μg protein *per* lane) and transferred to a nitrocellulose membrane for immunoblot analysis. The membrane was blocked with 5% skim milk in Tris Buffered Saline (TBS, pH 7.4) and incubated at 4°C overnight with primary antibodies against CYP1A2, CYP3A1, and GAPDH. After washing with TBS containing 0.1% TWEEN-20 (TBST), the membrane was incubated with an AP-linked secondary antibody at 25°C. The membrane was washed with TBST and incubated with AP chromogen (BCIP/NBT) system to detect the interested protein bands and photographed using UVP ChemStudio (Analytik Jena, Germany). Intensity analysis of protein bands was performed using Image J software (NIH). The housekeeping control, GAPDH, was used for protein level normalization.

#### Effects of formulated AL on CYP1A2 and CYP3A1 activities

Phenacetin and nifedipine, the substrates of CYP1A2 and CYP3A1, respectively, were used for investigation of the modulatory effects of AL on both enzymes [14]. The concentrations of rat liver microsomal proteins used in the incubation reaction were 0.5 and 0.3 mg/mL for CYP1A2 and CYP3A1, respectively (linear range of optimized protein concentration). The concentrations of phenacetin and nifedipine used were 20 μM and 40 μM, respectively.

The incubation mixture (500 μL) containing rat liver microsome (in 0.1 M potassium phosphate buffer, pH 7.4) and selective substrate was pre-incubated at 37°C for 5 min. After the addition of 1 mM NADPH, the reaction was further incubated for 60 and 40 min for CYP1A2 and CYP3A1 activities, respectively. Cold methanol (450 μL) was added to terminate the reaction, and 50 μL of caffeine and diazepam (the internal standard for CYP1A2 and CYP3A1, respectively) were added. After centrifugation (12,000x*g*, 15 min, 4°C), 20 μL of the supernatant were injected onto HPLC-UV for determination of paracetamol (CYP1A2 metabolite) and dehydronifedipine (CYP3A1 metabolite) concentrations. The HPLC system consisted of 1260 Quaternary pump VL, equipped with a 1260 ALS autosampler, 1260 DAD VL detector (Agilent Technologies), and OpenLAB CDS Software (version C.01.04). The column used for paracetamol and dehydronifedipine separation was a reversed-phase C-18 column (Thermo Hypersil Gold, 210 × 4.6 mm, 5 μm). The mobile phase for paracetamol determination consisted of water and methanol running at the gradient ratio of 20% methanol for 1 min, 45% methanol for 8 min, and 20% methanol for 7 min. The flow rate and UV detection were 1.0 mL/min and 240 nm, respectively.

The mobile phase for dehydronifedipine determination consisted of water and methanol (30%:70% v/v) running at a flow rate of 1.0 mL/min. UV detection was at 235 nm. The calibration curve concentrations for both CYP450-mediated metabolites was in the range of 1–50 μM, with correlation coefficients (r^2^) greater than 0.995. Quality control samples at low, medium and high concentrations were run together with each analytical batch. All analyses lay within 100±15% of the nominal values.

### Pharmacokinetic study

#### Treatment and blood sample collection

The pharmacokinetic study was conducted in three male WT rats following daily long-term multiple doses of 5,000 mg/kg body weight AL formulation for 9 months. Blood samples were collected from the tail vein at 0, 0.5, 1, 1.5, 2, 2.5, 3, 4, 6, and 8 hours after the last dose. Plasma was immediately separated through centrifugation at 3,000*xg* for 10 min (4°C).

#### Determination of ATD concentration

ATD was used as an active marker for the pharmacokinetic investigation of the formulated AL. Liquid-liquid extraction was applied for sample preparation. Briefly, the aliquot of pooled plasma from all rats (500 μL) was mixed with 10 μL of internal standard, 1,8-dihydroxyantraquinone. Protein contents were precipitated by adding 1 mL of acetonitrile and thoroughly mixed. After centrifugation (5,000*xg*, 20 min), the supernatant was transferred to a 15 mL tube, and 2 mL of dichloromethane was added and thoroughly mixed on a rotator for 30 min. The organic layer (lower layer) was transferred to a new tube and dried under nitrogen steam at room temperature.

Plasma concentrations of ATD in all samples were measured using HPLC according to the previously described method [15]. The HPLC system used was the same as previously described for CYP1A2 and CYP3A1 activities. The residue was reconstituted with 100 μL of acetonitrile and filtered through a 0.22 μm nylon filter membrane, and a 40 μL aliquot was subjected to the column. The mobile phase consisted of water and acetonitrile with a ratio of 30%:70% v/v, running at the flow rate of 1.0 mL/min. UV detection was set at 340 nm. The calibration curve (2.5–500 ng/mL) showed linearity with correlation coefficients (r^2^) greater than 0.995. Quality control samples at low, medium and high concentrations were run together with each analytical batch. All analyses lay within 100±15% of the nominal values.

The pharmacokinetic parameters of ATD were estimated using a non-compartmental model (Phoenix/WinNonlin version 8.3, Pharsight Corporation. 2016, Cary, North Carolina, USA). Maximum drug concentration in plasma (C_max_) and time to C_max_ (t_max_) were directly obtained from plasma concentration-time data. The total area under the plasma concentration-time curve (AUC_0−∞_) was calculated using the trapezoidal rule. The extrapolated AUC of the last sampling point to infinity was estimated by C_T_/elimination rate constant (λ_z_). The extrapolation was less than 5% of total areas in all cases. The total area under the first moment curve of the plasma concentration-time profile from time zero to infinity (AUMC_0-∞_) were determined, and mean residence time (MRT) was calculated from the ratio of AUMC_0-∞_/AUC_0−∞_.

### Biodistribution of ATD-PLGA-NPs

#### Preparation of ATD-PLGA-NPs and determination of ATD encapsulation and loading efficiencies

ATD-PLGA-NPs was formulated by solvent displacement method [16]. The encapsulation efficiency (%EE) and loading efficiency (%LE) were determined according to the protocol described in S3 Appendix.

#### Treatment and preparation of liver tissues

A male mouse was treated with a single dose of ATD-PLGA-NPs equivalent to 100 mg/kg body weight of ATD. The mouse was sacrificed under CO_2,_ and the liver sample was collected after 4 hours of dosing. The sample was rinsed with cold normal saline and sectioned (20 μm width) at -20°C using Cryostat Microtome (CM1950, Leica, Germany) and placed on ITO-coated glass slide. The cut tissue was stained with α-cyano-4-hydroxycinnamic acid under iMLayer (Shimadzu, Japan). The corresponding control mouse was treated with free ATD at the same dose.

#### MALDI-IMS analysis

MS images of the distribution of ATD in mouse liver were investigated using Imaging Mass Microscope (IMS) (iMScope TRIO, Shimadzu, Japan) in positive mode with a pitch of 36 μm for both x and y direction and mass to charge ratio range of 180–185, at a laser frequency of 1,000 Hz with 100 of shots. The sample and detector voltages were set at 3.50 kV and 2.00 kV, respectively, and the laser intensity was 15. The images of liver tissues were captured, and the amount of ATD in the liver was calculated and reported as intensity.

### Statistical analysis

SPSS software version 25 (IBM, NY, USA) was applied for all statistical analyses. The quantitative data are expressed as median (95% CI) for non-normally distributed data. The differences between each treatment and control or placebo groups were analyzed using the Mann-Whitney U test. The statistical significance level was set at ⍺ = 0.05 for all tests.

## Results

### Modulatory effects of formulated AL on CYP1A2 and CYP3A1 expression and activities

#### Effects of formulated AL on CYP1A2 and CYP3A1 mRNA expression

**Fig 1:**
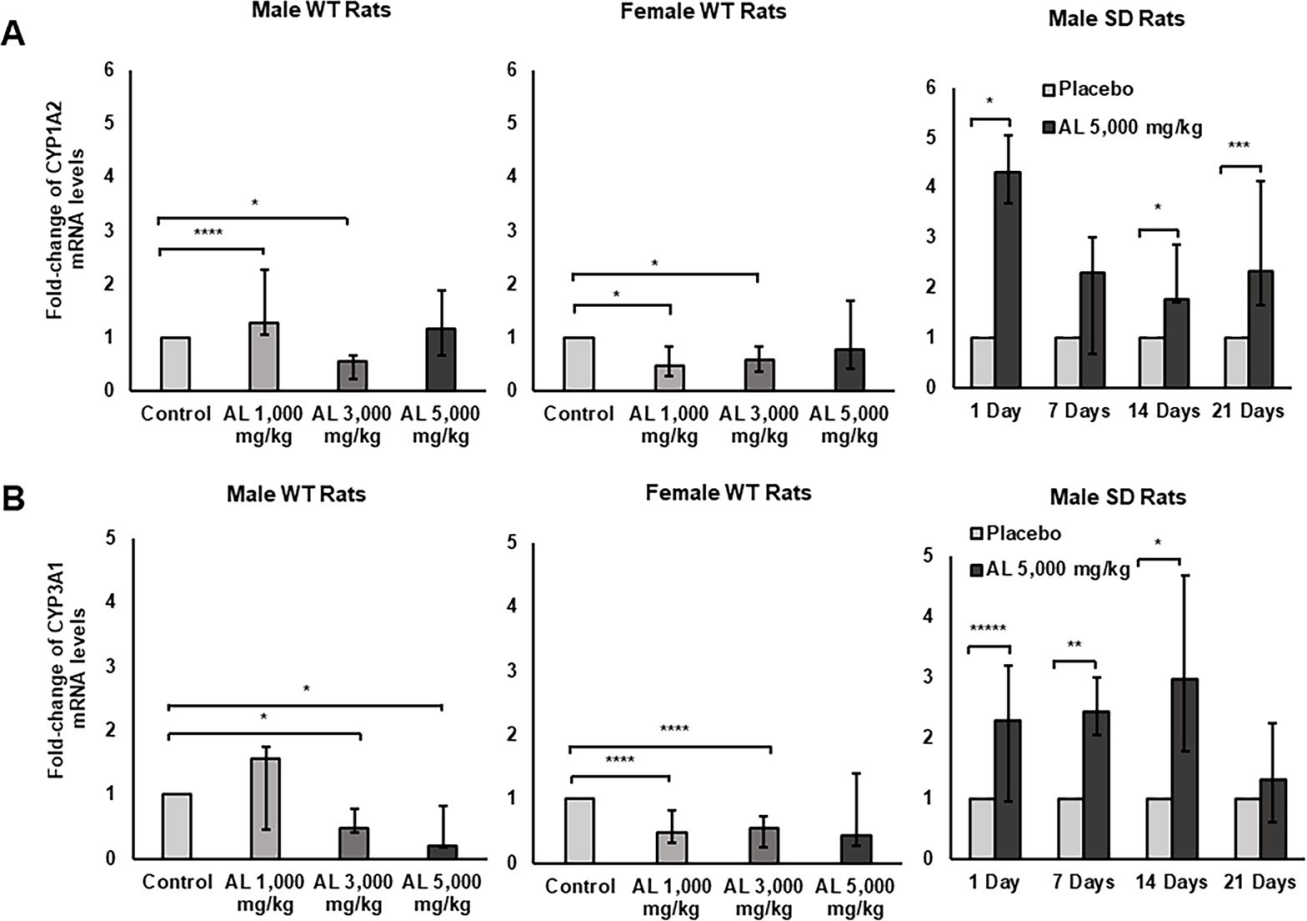
Fold-changes of CYP1A2 and CYP3A1 mRNA expression levels in rat livers. CYP1A2 (A) and CYP3A1 (B) mRNA expression in rat livers was investigated in (i) male and female WT rats after the administration of 1,000 (low dose), 3,000 (medium dose), 5,000 (high dose) mg/kg body weight/day of formulated AL for 12 months and (ii) male SD rats after the administration of 5,000 mg/kg body weight/day of placebo or formulated AL for 1,7, 14, and 21 days. Data are expressed as median (95% CI) from 3 rats, triplicate each. *p<0.001, **p = 0.001, ***p = 0.002, ****p = 0.003, *****p = 0.005 compared to placebo or control.

The fold-change of CYP1A2 and CYP3A1 mRNA expression levels in the livers of rats after long- and short-term administration of the formulated AL are presented in Fig 1A and 1B with S1 Table. For long-term exposure (Experiment I), AL showed dose-independent modulatory effects on CYP1A2 and CYP3A1 mRNA expression in both male and female rats. Short-term exposure of AL (Experiment II) on the other hand, upregulated the expression of both genes.

#### Effects of formulated AL on CYP1A2 and CYP3A1 protein expression

**Fig 2:**
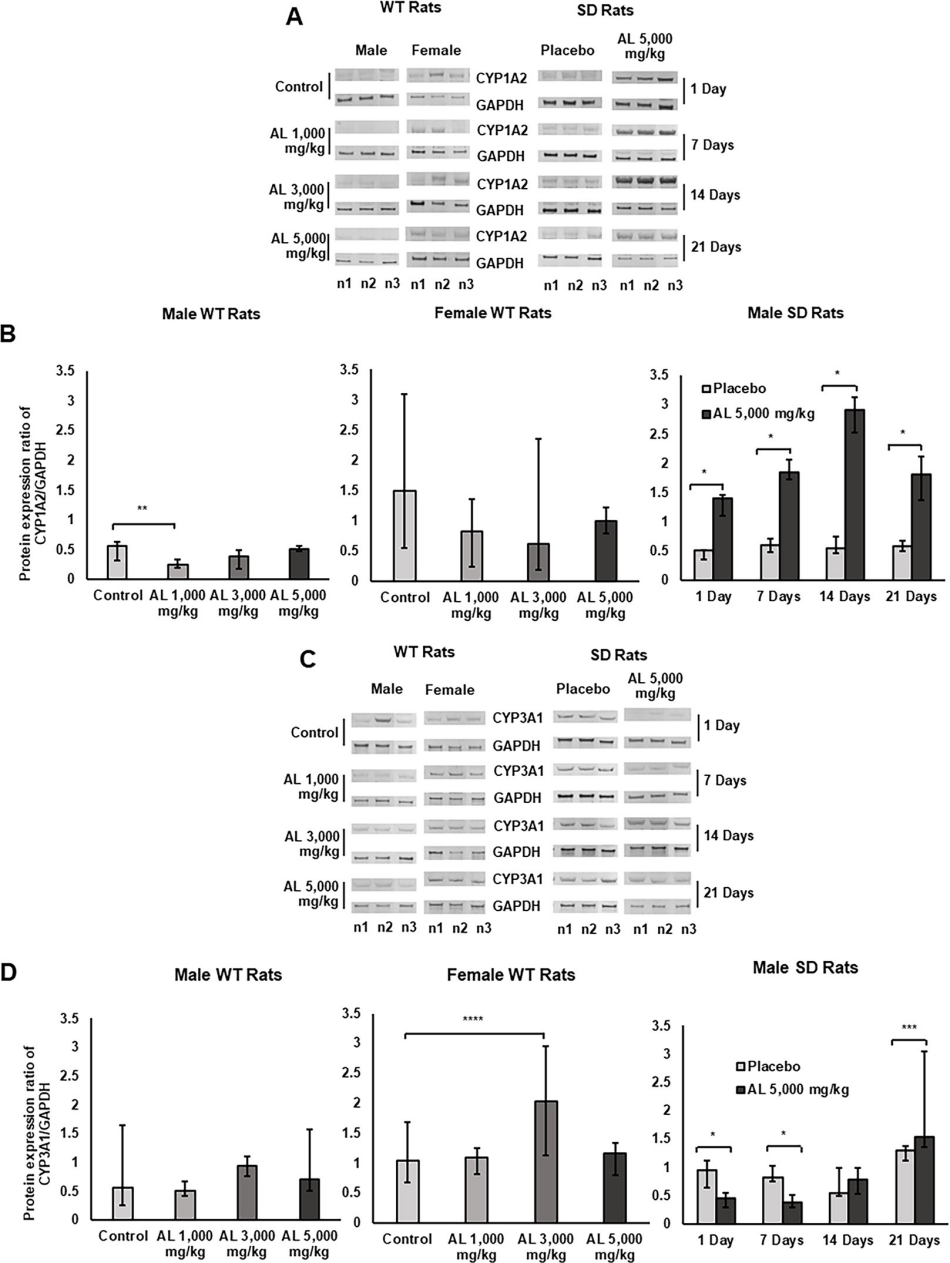
Western immunoblots analysis and protein expression ratio of CYP1A2 and CYP3A1 in rat livers. The analysis of western immunoblots and protein expression ratio of CYP1A2 (A-B) and CYP3A1 (C-D) were investigated in the livers of (i) male and female WT rats following exposure to 1,000 (low dose), 3,000 (medium dose), 5,000 (high dose) mg/kg body weight/day of formulated AL for 12 months, and in (ii) male SD rats following exposure to 5,000 mg/kg body weight/day of placebo or formulated AL for 1,7, 14, and 21 days. Data are expressed as median (95% CI) from 3 rats, triplicate each. *p<0.001, **p = 0.005, ***p = 0.009, ****p = 0.019 compared to placebo or control.

The western immunoblots analysis and protein expression ratio of CYP1A2 and CYP3A1 in the livers of rats after long- and short-term administration of formulated AL are shown in Fig 2A–2D with S2 Table. For long-term exposure (*Experiment I*), AL formulation downregulated CYP1A2 and upregulated CYP3A1 protein expressions in dose-independent manner. For short-term exposure (*Experiment II*), upregulation of CYP1A2 protein expression was observed in all rats that received the AL formulation. In rats treated with AL for 21 days, only the upregulation of CYP3A1 protein expression was found.

#### Effects of formulated AL on CYP1A2 and 3A1 activities

**Fig 3:**
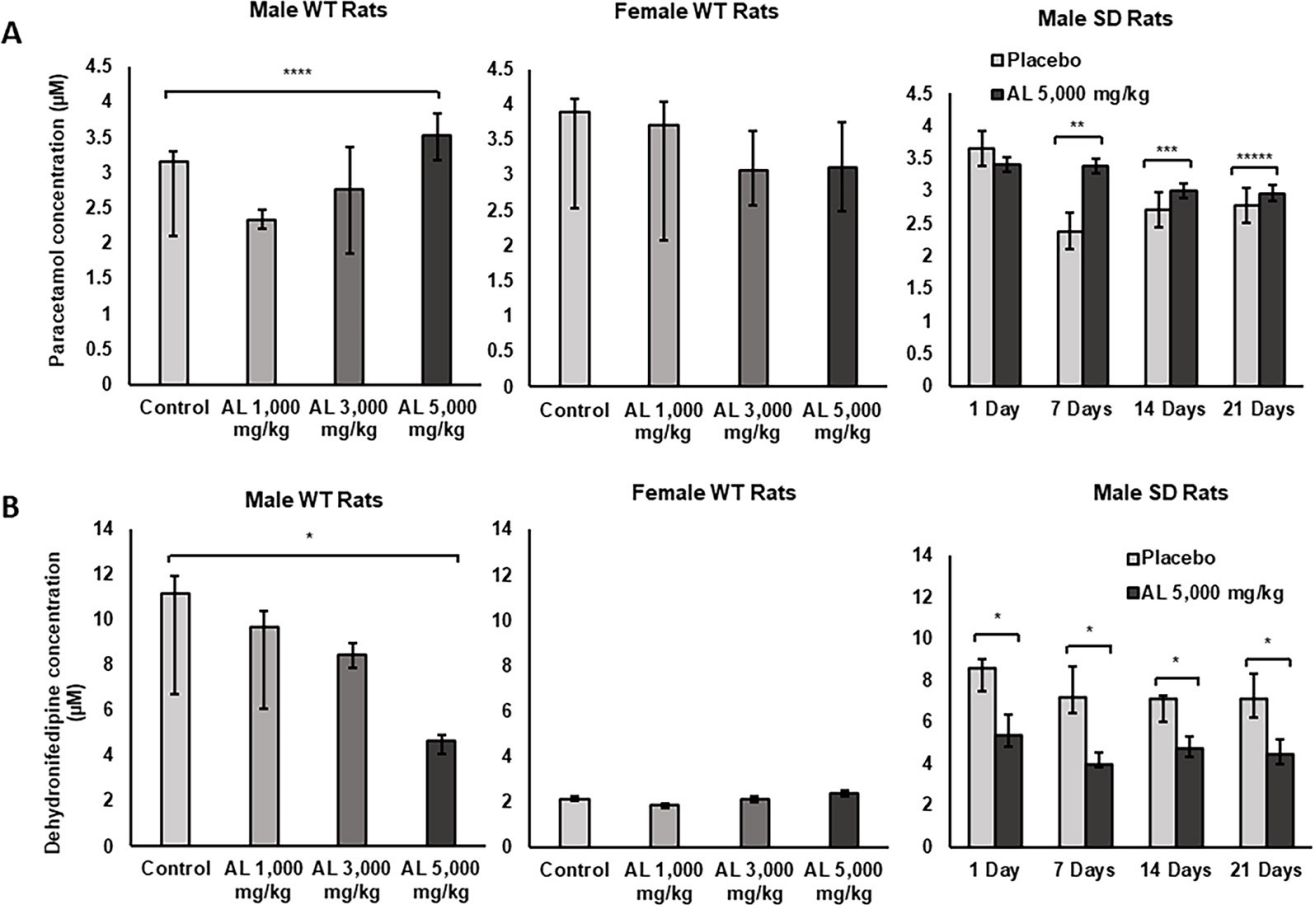
The CYP1A2- and CYP3A1-mediated metabolite in rat liver microsomes. CYP1A2 (A) and CYP3A1 (B) enzyme activities were determined in the liver microsomes of (i) male and female WT rats following exposure to 1,000 (low dose), 3,000 (medium dose), and 5,000 (high dose) mg/kg body weight/day of formulated AL for 12 months and (ii) male SD rats following exposure to 5,000 mg/kg body weight/day of placebo or formulated AL for 1,7, 14, and 21 days. Data are expressed as median (95% CI) from 3 rats, triplicate each. *p<0.001, **p = 0.002, ***p = 0.009, ****p = 0.010, *****p = 0.012 compared to placebo or control.

Paracetamol (CYP1A2-mediated metabolite) and dehydronifedipine (CYP3A1-mediated metabolite), were respectively used as markers of CYP1A2 and CYP3A1 activities in rat livers. The formation of paracetamol and dehydronifiedipine in the liver microsomes from rats following long- and short-term exposure to the formulated AL is presented in Fig 3A and 3B and S3 Table. For the long-term exposure (*Experiment I*), a significant increase in paracetamol formation (compared to control) was observed in male rats that received 5,000 mg/kg body weight AL (*p* = 0.010). In contrast, a significantly decrease in the level of dehydronifedipine (*p* < 0.001) was observed in this group of rats. The extent of paracetamol and dehydronifedipine formation in the livers of rats of both genders in other groups were comparable. For short-term exposure (*Experiment II*), a significant increase in paracetamol formation was found in rats that received the high daily doses of 5,000 mg/kg body weight AL for 7 (*p* = 0.002), 14 (*p* = 0.009), and 21(*p* = 0.012) days. A significant decrease in dehydronifedipine formation (*p* < 0.001) was found in rats treated with the high daily doses of 5,000 mg/kg body weight AL for all periods (1, 7, 14, and 21 days).

### Pharmacokinetic study

**Fig 4:**
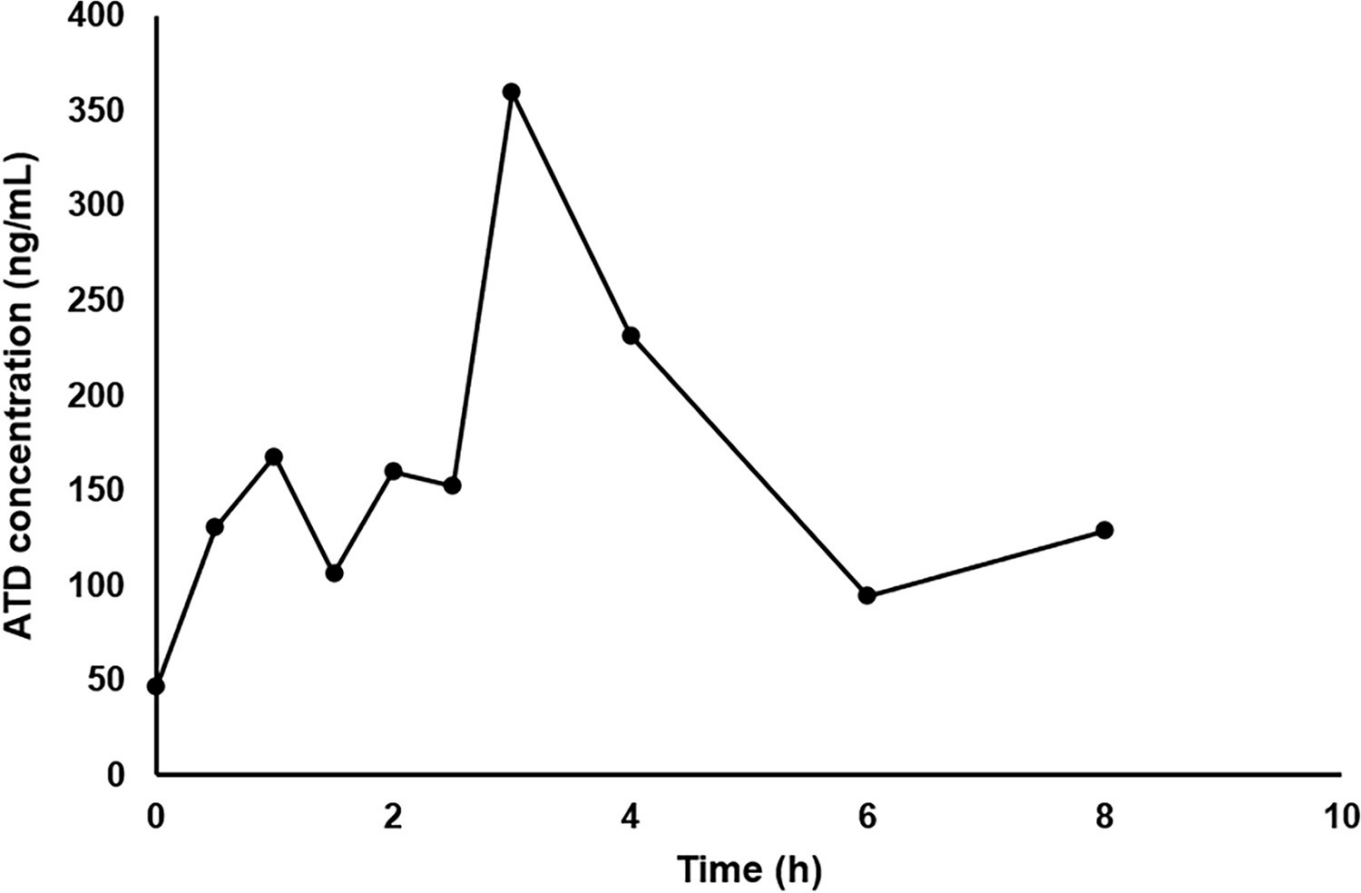
Plasma time-concentration profile of ATD after multiple oral daily administration of 5,000 mg/kg body weight of formulated AL in male WT rats.

ATD, an active compound of *A*. *lancea*, was used as a marker for formulated AL pharmacokinetic investigation. The plasma concentration-time profile and pharmacokinetic parameters of ATD after multiple daily doses of 5,000 mg/kg body weight for 9 months are presented in Fig 4 and Table 2, respectively. The residual amount of ATD from the previous dose was observed at 50 ng/mL. ATD was slowly absorbed, reaching the maximum level in plasma after 3 hours of administration. Complete elimination was at 6 hours.

**Table 2:** Pharmacokinetic parameters of ATD after oral administration of the daily dose of 5,000 mg/kg body weight formulated AL in male WT rats (pooled sample of three rats).

Parameter	
**C**_**max**_ **(ng/ml)**	359.73
**t**_**max**_ **(h)**	3
**λ**_**z**_ **(/h)**	1.23
**t**_**1/2z**_ **(h)**	0.56
**AUC**_**0−∞**_ **(ng.h/ml)**	896
**MRT (h)**	3.03

### Biodistribution of ATD-PLGA-NPs

#### Determination of ATD encapsulation and loading efficiencies

ATD-PLGA-NPs was successfully prepared with the high encapsulation and loading efficiencies [median (range) of 80.64 (78.33–81.39) and 3.20 (3.13–3.26) for %EE and %LE, respectively].

#### MALDI-IMS analysis

**Fig 5:**
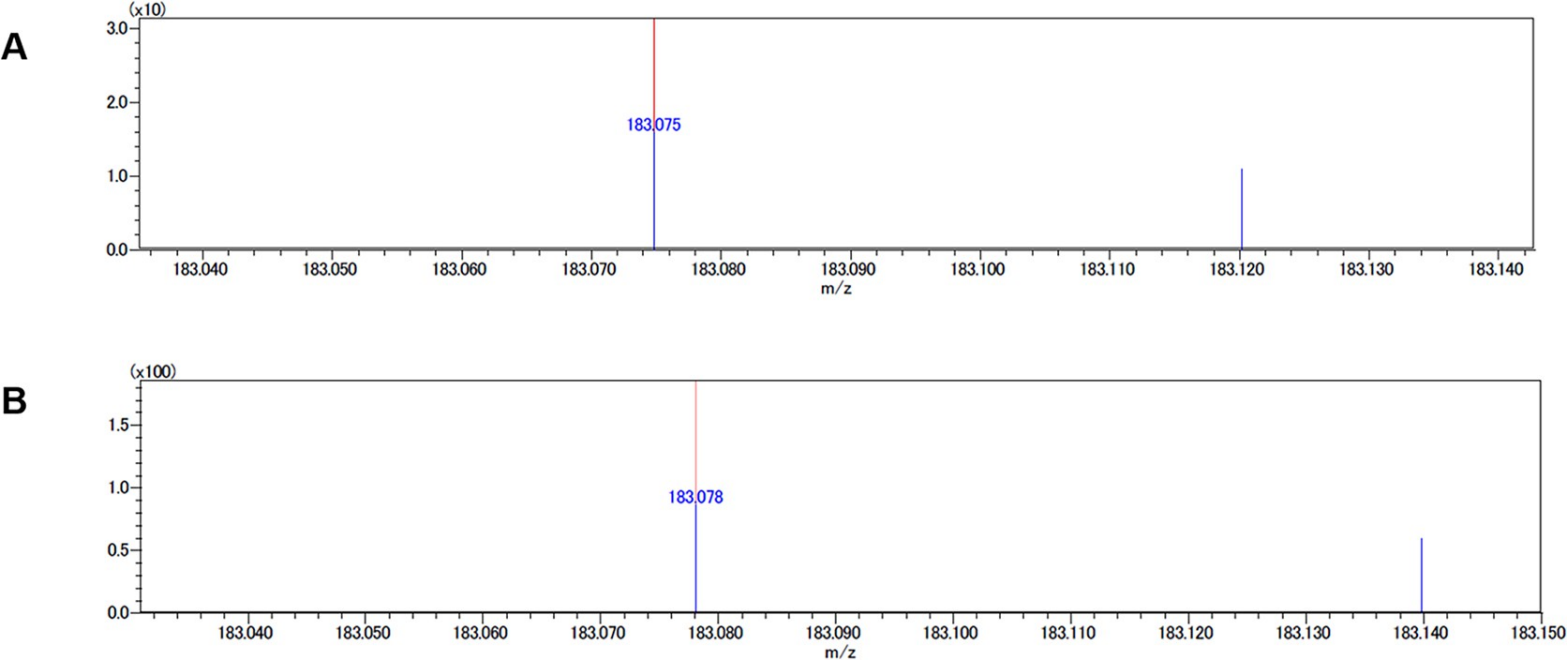
The MALDI mass spectra of ATD. MALDI mass analysis of ATD was performed in the mouse livers after being treated with a single dose of (A) free ATD and (B) ATD-PLGA-NPs at the dose of 100 mg/kg body weight of ATD.

**Fig 6:**
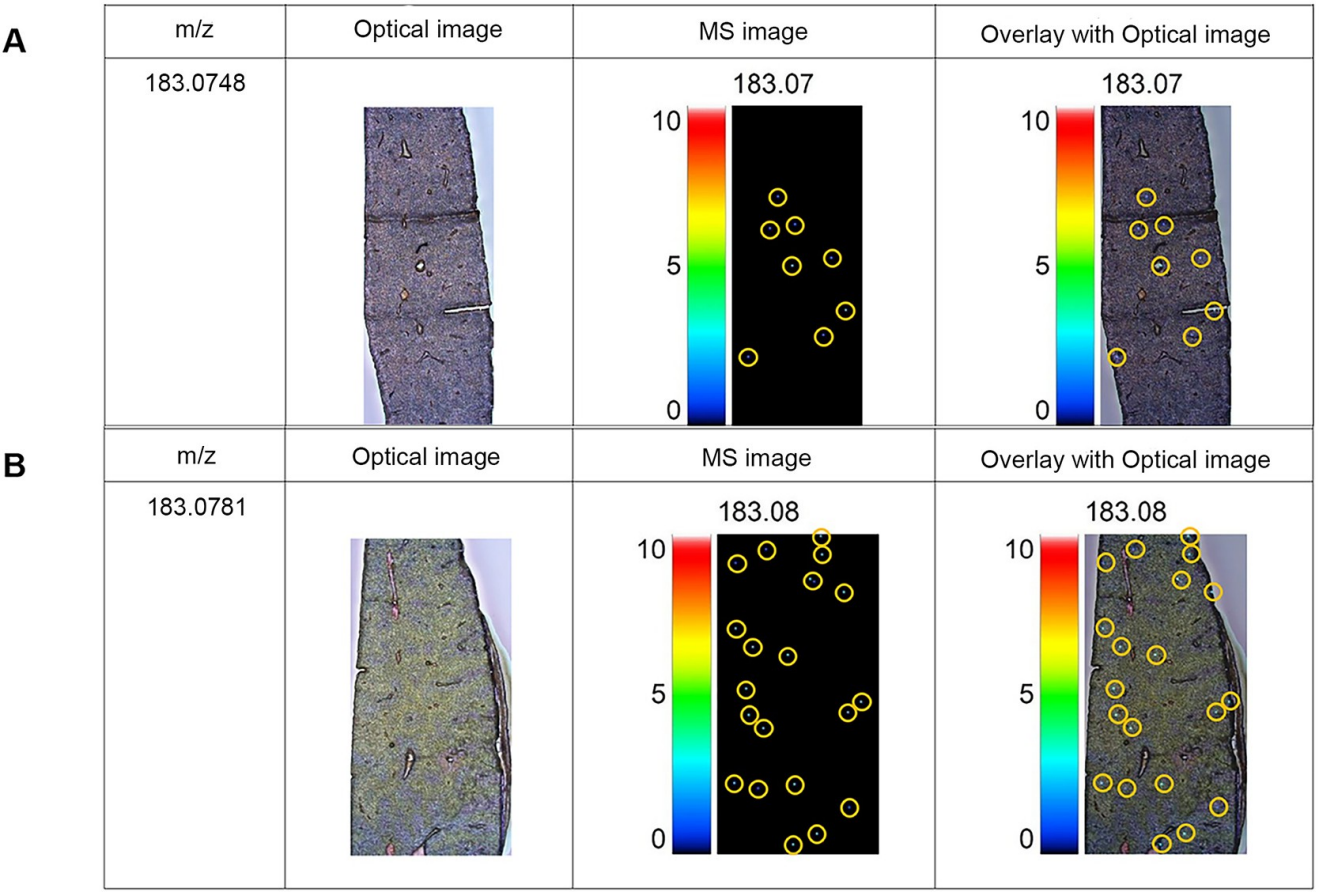
The MALDI imaging analysis of ATD. MALDI images of ATD were captured from the mouse livers after being treated with a single dose of (A) free ATD and (B) ATD-PLGA-NPs at the dose of 100 mg/kg body weight of ATD.

MALDI-IMS was used to analyze the localization of ATD in mouse liver tissues following the administration of free atractylodin (free ATD) and ATD-PLGA NPs. The mass spectra of ATD from both free ATD and ATD-PLGA NPs are shown in Fig 5. The m/z spectra of free ATD and ATD-PLGA-NPs were 183.0748 and 183.0781, respectively. From the MS images, ATD-PLGA-NPs showed about 5 times the higher intensity of ATD in mouse liver tissue compared with free ATD (87 and 16, respectively). The MALDI imaging analysis of both formulations is presented in Fig 6.

## Discussion

The dose levels of formulated AL used for the investigation of CYP450 modulatory activities and pharmacokinetic studies (1,000, 3,000, and 5,000 mg/kg body weight) and biodistribution study (100 mg/kg body weight of ATD) were selected based on the maximum tolerated dose (MTD) and no observed adverse effect (NOAEL) levels reported from the subacute toxicity studies [12, 13, 18].

CYP3A4 is the CYP450 isoform that contributes to the metabolism of 50% of the marketed drugs. In addition, the rat liver contains the CYP450 isoenzymes which are clinically relevant to human CYP450, including CYP1A and CYP3A [20, 21]. The potent inhibitory effect of AL extract on human CYP1A2 was shown in our previous study [14]. For these reasons, rat liver CYP1A2 and CYP3A1 isoforms (homologs to human CYP1A2 and CYP3A4, respectively) were selected for investigation of the modulatory effects of the formulated AL on CYP450 metabolizing enzymes in the current study. Long-term modulatory effects of the formulated AL on CYP1A2 and CYP3A1 expression (mRNA and proteins) and activities were initially investigated in male and female WT rat livers following exposure to the formulated AL at the daily oral doses of 1,000 (low dose), 3,000 (medium dose), and 5,000 (high dose) mg/kg body weight for 12 months. Results showed both inducing and inhibitory effects of AL on the expression of CYP1A2 and CYP3A1 mRNA and proteins in male and female rats in a dose-independent manner. For CYP450 enzyme activities, male rats receiving formulated AL at the high daily dose level of 5,000 mg/kg body weight showed significant induction of CYP1A2, while inhibition of CYP3A1. Lower dose levels of the formulated AL (1,000 and 3,000 mg/kg body weight) did not significantly inhibit nor induce both CYP1A2 and CYP3A1 activities in the livers of both male and female rats. This suggests the dose-dependent inducing or inhibitory effect of AL on the activity of both enzymes. The observed lack of correlation between the modulatory effects on mRNA and protein expression and enzyme activities may suggest direct interference of AL with the enzyme activity. The results also provide evidence of sex differences in the modulatory effects of AL. It was noted, however, that, although the results of the phase I study reported a nonsignificant difference in pharmacokinetic parameters of ATD between male and female subjects, C_max_ and AUC were slightly higher in females particularly after multiple dose administration [15]. The modulatory effect of formulated AL at the high dose level of 5,000 mg/kg body weight was further investigated in male SD rats following short-term exposure. Interestingly, results showed similar findings as that were found following long-term exposure, *i*.*e*., inhibitory effect on CYP3A1 but inducing effect on CYP1A2 activity. It was noted, however, for the inhibitory effect on CYP1A2 activity in rats treated with formulated AL for 1 day, similarly to that observed in the previous *in vitro* study [14]. This suggests a time-dependent inducing or inhibitory effect of the formulated AL on CYP450 activity. Since CYP1A2 activity was well correlated with mRNA and protein expression, the change in the activity of this enzyme is likely to be a result of an increase in mRNA and protein expression, but not the direct interference of AL with the enzyme activity. In contrast, the activity of CYP3A1 was not correlated with both mRNA and protein expression, which may indicate direct interference of AL with the enzyme activity. The current investigation of CYP450 modulatory activities was a part of a large study to evaluate chronic toxicity (long-term exposure) and to identify the metabolic pathway (short-term exposure) of the formulated AL. Different strains of rats were, therefore, used in the current study to avoid unnecessary inclusion of additional animals for the study. Hepatic CYP1A and CYP3A were shown to be prevailing expressed in WT rats compared with SD rats [22]. However, this should not affect the results and interpretation as the comparison was made with control or placebo.

Human CYP1A2 metabolizes various drugs, *e*.*g*., phenacetin, tacrine, ropinirole, acetaminophen, theophylline, caffeine, and various pre-carcinogens, *e*.*g*., benzo[*a*]pyrene, and aflatoxin B_1_ [20, 23, 24]. Induction in CYP1A2 was shown to enhance the activation of pre-carcinogen to a carcinogen [23]. CYP1A2 induction is under the transcriptional regulation of the aryl hydrocarbon receptor (AhR). The process of induction involves the interaction of AhR/ARNT (aryl hydrocarbon receptor nuclear translocator) complex with upstream enhancer components. This is followed by the transmission of induction signal to promotor and subsequent transcription of mRNA and translocation to corresponding protein [25, 26]. CYP3A4 is liable for the biotransformation of several drugs; some of them, *e*.*g*. carbamazepine, cyclosporine, digoxin and tacrolimus, are drugs with narrow therapeutic index values [27]. Although rat CYP1A2 and CYP3A1 are orthologues of human CYP1A2 and CYP3A4, differences in inducing and inhibitory effects and catalytic activities are possible.

The pharmacokinetic study in rats after long-term multiple daily dosing of formulated AL showed slightly prolonged t_max_ and MRT compared with that were observed in humans [15]. This suggests slower absorption of ATD from the gastrointestinal tract of rats. ATD was rapidly eliminated within 6 hours of dosing in both rats and humans [15]. Improvement in the bioavailability and biodistribution of ATD has been well demonstrated when ATD was formulated as ATD-PLGA-NPs [16]. The prepared ATD-PLGA-NPs showed comparable %EE and %LE compared with that reported in the previous study. The time of liver sample collection was selected based on the information provided in the previous study showing the maximum level of distribution of ATD-PLGA-NPs to the liver at 4 hours after administration [16]. MALDI-IMS analysis confirmed a relatively high extent of ATD distribution when administered as ATD-PLGA-NPs compared with unencapsulated ATD. This could be due to the improved bioavailability of ATD-PLGA-NPs. The nanoparticle size is sufficiently small to be absorbed and distributed to the target site. MALDI-IMS has become a widely used method for molecular tissue and morphological analysis due to high sensitivity, wide range of molecules, and molecular specificity [28]. The study was the first that investigated the localization of ATD in mice livers using MALDI-IMS.

Formulated AL and ATD are potential candidates and are currently under research and development for cholangiocarcinoma treatment [8, 16, 29]. Phase I clinical trial of AL showed promising safety and pharmacokinetic profiles [15], as well as immunomodulatory activity in healthy Thai subjects [30]. Phase II dose-finding study is currently in progress to find the most effective dose in patients with advanced stage cholangiocarcinoma. The results of the current study thus provide important information to aid the design of an optimal dose regimen of the formulated AL for cholangiocarcinoma patients. Clinical use of the maximum dose of 5,000 mg/kg body weight should be of concern for the potential of metabolic interactions with therapeutic drugs currently co-administered with AL, particularly those metabolized by CYP1A2 and CYP3A4. This is of concern, considering the requirement for long-term administration of AL in patients with cholangiocarcinoma. Moreover, the results of pharmacokinetic investigation after long-term administration could support long-term use in humans. The ATD-PLGA-NPs formulation could be a promising drug delivery system to improve the bioavailability of ATD at the target sites of action in cholangiocarcinoma patients.

## Conclusions

The formulated AL at the highest dose level of 5,000 mg/kg body weight produced inducing and inhibitory effects on CYP1A2 and CYP3A1 in the livers of male rats following short- (up to 21 days) and long-term (12 months) treatment. The low dose levels did not inhibit or induce the activities of both enzymes in male and female rats. These low dose levels should be considered for clinical use of AL to avoid potential herb-drug interaction. The ATD-PLGA-NPs nanoformulation is a potential drug delivery system for cholangiocarcinoma treatment.

## Supporting information

S1 Appendix Total RNA and cDNA synthesis. https://doi.org/10.6084/m9.figshare.21330819.
(DOCX)

S2 Appendix Preparation of liver microsomal protein. https://doi.org/10.6084/m9.figshare.21330828.
(DOCX)

S3 Appendix Preparation of ATD-PLGA-NPs and determination of ATD encapsulation and loading efficiencies. https://doi.org/10.6084/m9.figshare.21330834.
(DOCX)

S1 Table The fold-change of CYP1A2 and CYP3A1 mRNA expression levels in rat livers. CYP1A2 and CYP3A1 mRNA expression in rat livers was investigated in (i) Male and female WT rats after the administration of 1,000 (low dose), 3,000 (medium dose), 5,000 (high dose) mg/kg body weight/day of formulated AL for 12 months and (ii) male SD rats after the administration of 5,000 mg/kg body weight/day of placebo or formulated AL for 1,7, 14, and 21 days. https://doi.org/10.6084/m9.figshare.21330840.
(DOCX)

S2 Table Protein expression ratio of CYP1A2 and CYP3A1 in rat livers. The protein expression ratio of CYP1A2 and CYP3A1 in rat livers was performed in male and female WT rats after the administration of 1,000 (low dose), 3,000 (medium dose), 5,000 (high dose) mg/kg body weight/day of formulated AL for 12 months and male SD rats after the administration of 5,000 mg/kg body weight/day of placebo or formulated AL for 1,7, 14, and 21 days. https://doi.org/10.6084/m9.figshare.21330846.
(DOCX)

S3 Table The CYP1A2- and CYP3A1-mediated metabolite in rat liver microsomes. CYP1A2 and CYP3A1 enzyme activities were evaluated in the liver microsomes of (i) male and female WT rats after the administration of 1,000 (low dose), 3,000 (medium dose), 5,000 (high dose) mg/kg body weight/day of formulated AL for 12 months and (ii) male SD rats after the administration of 5,000 mg/kg body weight/day of placebo or formulated AL for 1,7, 14, and 21 days. https://doi.org/10.6084/m9.figshare.21330852.
(DOCX)

S1 Raw images https://doi.org/10.6084/m9.figshare.21330858. (PDF)
