# Supplementary material for: Atractylodes lancea for cholangiocarcinoma: Modulatory effects on CYP1A2 and CYP3A1 and pharmacokinetics in rats and biodistribution in mice
Source: PLoS One. 2022 Nov 14;17(11):e0277614. doi: 10.1371/journal.pone.0277614 (PMC9662714; doi:10.1371/journal.pone.0277614)
Supplement: S1 Appendix — https://doi.org/10.6084/m9.figshare.21330819. (DOCX) [file pone.0277614.s001.docx]

**Total RNA and cDNA synthesis**

All rats were sacrificed under CO_2_ after 24 h of the last administration of formulated AL. Liver samples were isolated and washed with cold normal saline.  Trizol^TM^ reagent was used for RNA isolation following the manufacturer’s protocol. In brief, liver tissue (50-100 mg) was homogenized in Trizol^TM^ reagent and chloroform, followed by precipitation with isopropyl alcohol. RNA was separated and washed with 75% ethanol. After drying, RNA was dissolved in sterile DEPC-treated water and concentration was measured using NanoDrop. RNase-Free DNase was finally used to treat RNA products to degrade both single-stranded and double-stranded DNA following the manufacturer’s instructions.

Complementary DNA (cDNA) was produced using SuperScript^TM^ III Reverse Transcriptase following the manufacturer’s protocol. In brief, RNA (10 pg - 5 µg) were incubated with oligo(dT)_20_, dNTP mix, and DEPC-treated water. The mixture was further incubated with DNA Synthesis Mix and RNase H, respectively. cDNA products were stored at -20 °C for further use.
