# Supplementary material for: Atractylodes lancea for cholangiocarcinoma: Modulatory effects on CYP1A2 and CYP3A1 and pharmacokinetics in rats and biodistribution in mice
Source: PLoS One. 2022 Nov 14;17(11):e0277614. doi: 10.1371/journal.pone.0277614 (PMC9662714; doi:10.1371/journal.pone.0277614)
Supplement: S2 Appendix — https://doi.org/10.6084/m9.figshare.21330828. (DOCX) [file pone.0277614.s002.docx]

**Preparation of liver microsomal protein**

The liver sample (2-4 g) was homogenized with 0.05 M sodium phosphate buffer (pH 7.4) and 0.1 mM EDTA (3 times of liver volume) using Potter-Elvehjem Tissue Homogenizer and centrifuge at 9,000x*g* for 20 min (4 °C). The supernatant was ultracentrifuged (105,000x*g*, 60 min, 4 °C) twice. The pellets were homogenized with storage buffer (0.05 M potassium phosphate buffer pH 7.4, 0.1 mM EDTA, and 10% glycerol). The concentration of microsomal protein was measured using Pierce BCA protein assay kit and stored at -80 °C for further use.
