# Supplementary material for: Atractylodes lancea for cholangiocarcinoma: Modulatory effects on CYP1A2 and CYP3A1 and pharmacokinetics in rats and biodistribution in mice
Source: PLoS One. 2022 Nov 14;17(11):e0277614. doi: 10.1371/journal.pone.0277614 (PMC9662714; doi:10.1371/journal.pone.0277614)
Supplement: S3 Appendix — https://doi.org/10.6084/m9.figshare.21330834. (DOCX) [file pone.0277614.s003.docx]

**Preparation of ATD-PLGA-NPs and determination of ATD encapsulation and loading efficiencies**

The ATD (2 mg) and PLGA (50 mg) were dissolved in 2 mL of acetone and directly injected dropwise into 15 mL of 1% (w/v) poloxamer 407 and stirred at 550 *rpm* using a syringe pump (KD Scientific, USA). The nanoparticle products were centrifuged at 12,000*xg* for 10 min (4 °C) to remove the supernatant. The pellets were resuspended in sterile deionized water and stored at 4 °C until use. The encapsulation efficiency (%EE) and loading efficiency (%LE) was then determined. The suspension of ATD-PLGA-NPs (1 mL) was centrifuged at 12,000*xg* for 10 min (4 °C). After removing the supernatant, an equal amount of DMSO was added to dissolve the particles and sonicated until the solution became clear. ATD concentrations were measured spectrophotometrically using a UV reader (Thermo Fisher Scientific, MA, USA) at the wavelength of 340 nm. The percentages of encapsulation and loading efficiencies were calculated as the following equations:

$\%EE = (Amount of ATD loaded in NPs/Amount of ATD added) x 100\%$ (3)

$\%LE = (Amount of ATD loaded in NPs/Amount of NPs) x 100\%$ (4)
