# Supplementary material for: Atractylodes lancea for cholangiocarcinoma: Modulatory effects on CYP1A2 and CYP3A1 and pharmacokinetics in rats and biodistribution in mice
Source: PLoS One. 2022 Nov 14;17(11):e0277614. doi: 10.1371/journal.pone.0277614 (PMC9662714; doi:10.1371/journal.pone.0277614)
Supplement: S1 Table — CYP1A2 and CYP3A1 mRNA expression in rat livers was investigated in (i) Male and female WT rats after the administration of 1,000 (low dose), 3,000 (medium dose), 5,000 (high dose) mg/kg body weight/day of formulated AL for 12 months and (ii) male SD rats after the administration of 5,000 mg/kg body weight/day of placebo or formulated AL for 1,7, 14, and 21 days. https://doi.org/10.6084/m9.figshare.21330840. (DOCX) [file pone.0277614.s004.docx]

**S1 Table. The fold-change of CYP1A2 and CYP3A1 mRNA expression levels in rat livers**.

| WT Rats | | | SD Rats | | |
| --- | --- | --- | --- | --- | --- |
| Groups | **Median (95% CI)** | | **Groups** | **Median (95% CI)** | |
|  | **Male** | **Female** |  | **Placebo** | **AL 5,000 mg/kg** |
| Fold-change of CYP1A2 mRNA levels | | | | | |
| Control | 1 (1-1) | 1 (1-1) | **1 Day** | 1 (1-1) | 4.3 (3.67-5.05) * |
| AL 1,000 mg/kg | 1.28 (1.04-2.26) **** | 0.46 (0.28-0.82) * | **7 Days** | 1 (1-1) | 2.29 (0.68-2.99) |
| AL 3,000 mg/kg | 0.54 (0.21-0.66) * | 0.59 (0.37-0.81) * | **14 Days** | 1 (1-1) | 1.75 (1.71-2.85) * |
| AL 5,000 mg/kg | 1.16 (0.65-1.88) | 0.78 (0.42-1.69) | **21 Days** | 1 (1-1) | 2.33 (1.65-4.11) *** |
| Fold-change of CYP3A1 mRNA levels | | | | | |
| Control | 1 (1-1) | 1 (1-1) | **1 Day** | 1 (1-1) | 2.29 (0.95-3.18) ***** |
| AL 1,000 mg/kg | 1.55 (0.45-1.74) | 0.48 (0.31-0.82) **** | **7 Days** | 1(1-1) | 2.43 (2.03-2.99) ** |
| AL 3,000 mg/kg | 0.48 (0.41-0.77) * | 0.55 (0.25-0.72) **** | **14 Days** | 1 (1-1) | 2.96 (1.76-4.67) * |
| AL 5,000 mg/kg | 0.2 (0.17-0.82) * | 0.42 (0.28-1.4) | **21 Days** | 1 (1-1) | 1.3 (0.61-2.23) |

Data are expressed as median (95% CI) from 3 rats, triplicate each. **p*<0.001, ***p*=0.001, ****p*=0.002, *****p*=0.003, ******p*=0.005 compared to placebo (SD rats) or control (WT rats).
