# Supplementary material for: Atractylodes lancea for cholangiocarcinoma: Modulatory effects on CYP1A2 and CYP3A1 and pharmacokinetics in rats and biodistribution in mice
Source: PLoS One. 2022 Nov 14;17(11):e0277614. doi: 10.1371/journal.pone.0277614 (PMC9662714; doi:10.1371/journal.pone.0277614)
Supplement: S2 Table — The protein expression ratio of CYP1A2 and CYP3A1 in rat livers was performed in male and female WT rats after the administration of 1,000 (low dose), 3,000 (medium dose), 5,000 (high dose) mg/kg body weight/day of formulated AL for 12 months and male SD rats after the administration of 5,000 mg/kg body weight/day of placebo or formulated AL for 1,7, 14, and 21 days. https://doi.org/10.6084/m9.figshare.21330846. (DOCX) [file pone.0277614.s005.docx]

**S2 Table. Protein expression ratio of CYP1A2 and CYP3A1 in rat livers**.

| WT Rats | | | SD Rats | | |
| --- | --- | --- | --- | --- | --- |
| Groups | **Median (95% CI)** | | **Groups** | **Median (95% CI)** | |
|  | **Male** | **Female** |  | **Placebo** | **AL 5,000 mg/kg** |
| Protein expression ratio of CYP1A2 | | | | | |
| Control | 0.57 (0.31-0.63) | 1.49 (0.54-3.10) | **1 Day** | 0.52 (0.36-0.63) | 1.41 (1.10-1.45) * |
| AL 1,000 mg/kg | 0.24 (0.19-0.34) ** | 0.83 (0.23-1.35) | **7 Days** | 0.61 (0.49-0.79) | 1.85 (1.73-2.06) * |
| AL 3,000 mg/kg | 0.39 (0.17-0.50) | 0.61 (0.18-2.36) | **14 Days** | 0.56 (0.47-0.65) | 2.92 (2.53-3.13) * |
| AL 5,000 mg/kg | 0.51 (0.47-0.56) | 1 (0.79-1.21) | **21 Days** | 0.59 (0.50-0.73) | 1.81 (1.37-2.12) * |
| Protein expression ratio of CYP3A1 | | | | | |
| Control | 0.56 (0.25-1.64) | 1.05 (0.68-1.68) | **1 Day** | 0.96 (0.64-1.12) | 0.46 (0.29-0.55) * |
| AL 1,000 mg/kg | 0.51 (0.42-0.67) | 1.10 (1.25-0.81) | **7 Days** | 0.83 (0.75-1.03) | 0.38 (0.30-0.51) * |
| AL 3,000 mg/kg | 0.94 (0.76-1.11) | 2.03 (1.13-2.95) **** | **14 Days** | 0.55 (0.50-1.00) | 0.79 (0.53-0.99) |
| AL 5,000 mg/kg | 0.70 (0.50-1.57) | 1.16 (0.80-1.34) | **21 Days** | 1.30 (1.12-1.37) | 1.54 (1.36-3.05) *** |

The data are expressed as median (95% CI) from 3 rats, triplicate each. **p*<0.001, ***p*=0.005, ****p*=0.009, *****p*=0.019 compared to placebo (SD rats) or control (WT rats).
