# Supplementary material for: Atractylodes lancea for cholangiocarcinoma: Modulatory effects on CYP1A2 and CYP3A1 and pharmacokinetics in rats and biodistribution in mice
Source: PLoS One. 2022 Nov 14;17(11):e0277614. doi: 10.1371/journal.pone.0277614 (PMC9662714; doi:10.1371/journal.pone.0277614)
Supplement: S3 Table — CYP1A2 and CYP3A1 enzyme activities were evaluated in the liver microsomes of (i) male and female WT rats after the administration of 1,000 (low dose), 3,000 (medium dose), 5,000 (high dose) mg/kg body weight/day of formulated AL for 12 months and (ii) male SD rats after the administration of 5,000 mg/kg body weight/day of placebo or formulated AL for 1,7, 14, and 21 days. https://doi.org/10.6084/m9.figshare.21330852. (DOCX) [file pone.0277614.s006.docx]

**S3 Table. The CYP1A2- and CYP3A1-mediated metabolite in rat liver microsomes**.

| WT Rats | | | SD Rats | | |
| --- | --- | --- | --- | --- | --- |
| Groups | **Median (95% CI)** | | **Groups** | **Median (95% CI)** | |
|  | **Male** | **Female** |  | **Placebo** | **AL 5,000 mg/kg** |
| Paracetamol concentration (µM) | | | | | |
| Control | 3.15 (2.11-3.30) | 3.90 (2.54-4.09) | **1 Day** | 3.66 (2.88-3.90) | 3.41 (2.08-3.45) |
| AL 1,000 mg/kg | 2.33 (2.21-2.48) | 3.71 (2.08-4.05) | **7 Days** | 2.39 (2.26-3.16) | 3.39 (3.14-3.79) ** |
| AL 3,000 mg/kg | 2.76 (1.85-3.37) | 3.07 (2.58-3.63) | **14 Days** | 2.71 (2.54-2.81) | 3.01 (2.75-4.00) *** |
| AL 5,000 mg/kg | 3.52 (3.18-3.85) **** | 3.12 (2.50-3.75) | **21 Days** | 2.79 (2.58-2.95) | 2.97 (2.82-3.60) ***** |
| Dehydronifedipine concentration (µM) | | | | | |
| Control | 11.12 (6.70-11.90) | 2.17 (1.83-2.65) | **1 Day** | 8.58 (7.46-9.02) | 5.39 (4.85-6.39) * |
| AL 1,000 mg/kg | 9.65 (6.07-10.39) | 1.87 (1.24-2.24) | **7 Days** | 7.23 (6.42-8.70) | 3.99 (4.56-3.82) * |
| AL 3,000 mg/kg | 8.45 (7.89-8.97) | 2.14 (1.32-2.54) | **14 Days** | 7.15 (6.04-7.27) | 4.77 (4.32-5.34) * |
| AL 5,000 mg/kg | 4.66 (4.05-4.93) * | 2.40 (1.62-2.65) | **21 Days** | 7.14 (6.24-8.31) | 4.46 (3.95-5.19) * |

The data are expressed as median (95% CI) from 3 rats, triplicate each. **p*<0.001, ***p*=0.002, ****p*=0.009, *****p*=0.010, ******p*=0.012 compared to placebo (SD rats) or control (WT rats).
