# Supplementary material for: Atractylodes lancea for cholangiocarcinoma: Modulatory effects on CYP1A2 and CYP3A1 and pharmacokinetics in rats and biodistribution in mice
Source: PLoS One. 2022 Nov 14;17(11):e0277614. doi: 10.1371/journal.pone.0277614 (PMC9662714; doi:10.1371/journal.pone.0277614)

S1 Raw images

Fig 2A Western blot analysis of CYP1A2 in the livers of male and female WT rats treated with 1,000 (low dose), 3,000 (medium dose), 5,000 (high dose) mg/kg BW AL for 12 months. (Lanes in the boxes were cropped and presented in Fig 2A).

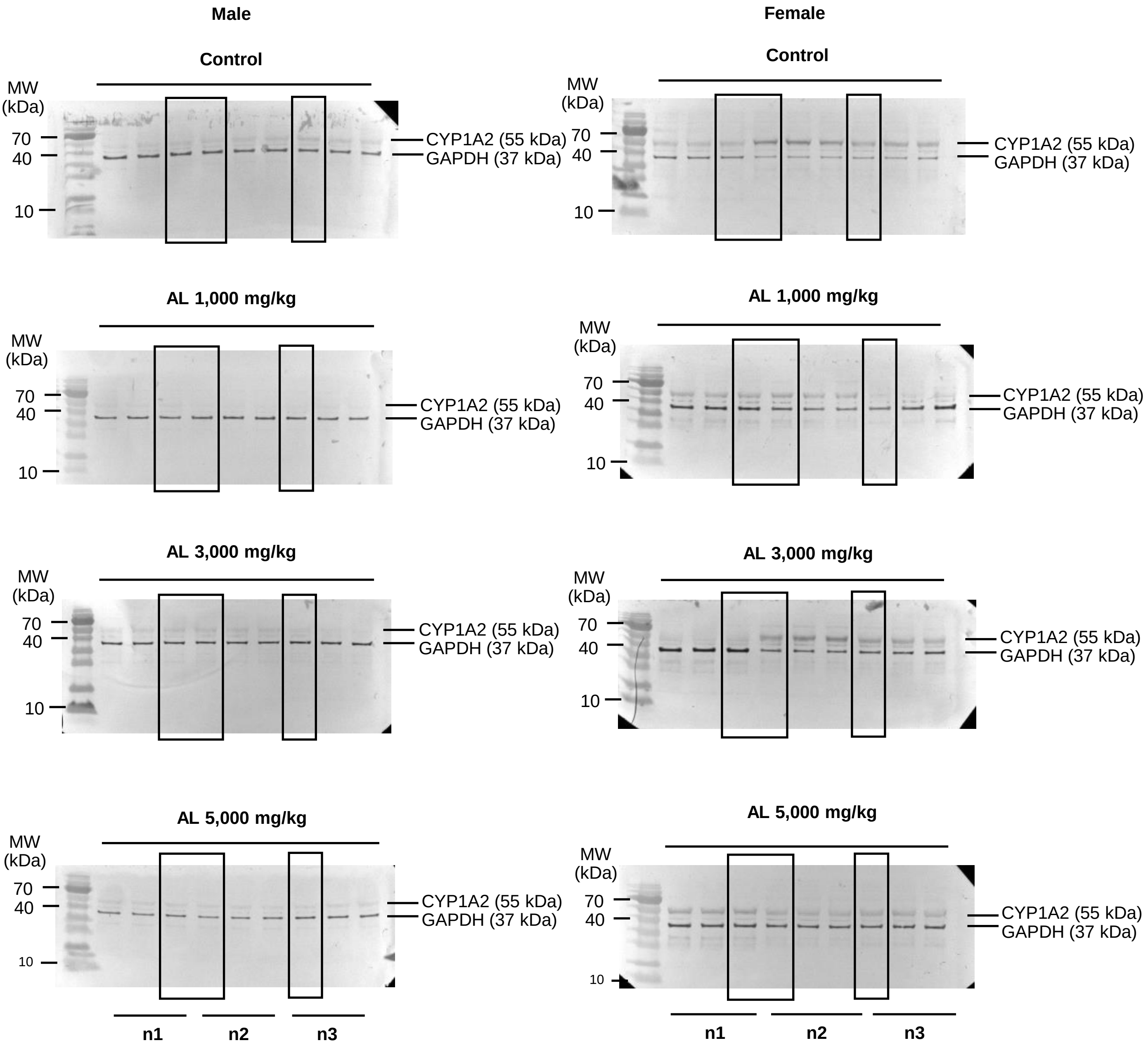

Fig 2A Western blot analysis of CYP1A2 in the livers of male SD rats treated with 5,000 mg/kg BW of placebo or AL for 1,7, 14, and 21 days. (Lanes in the boxes were cropped and presented in Fig 2A).

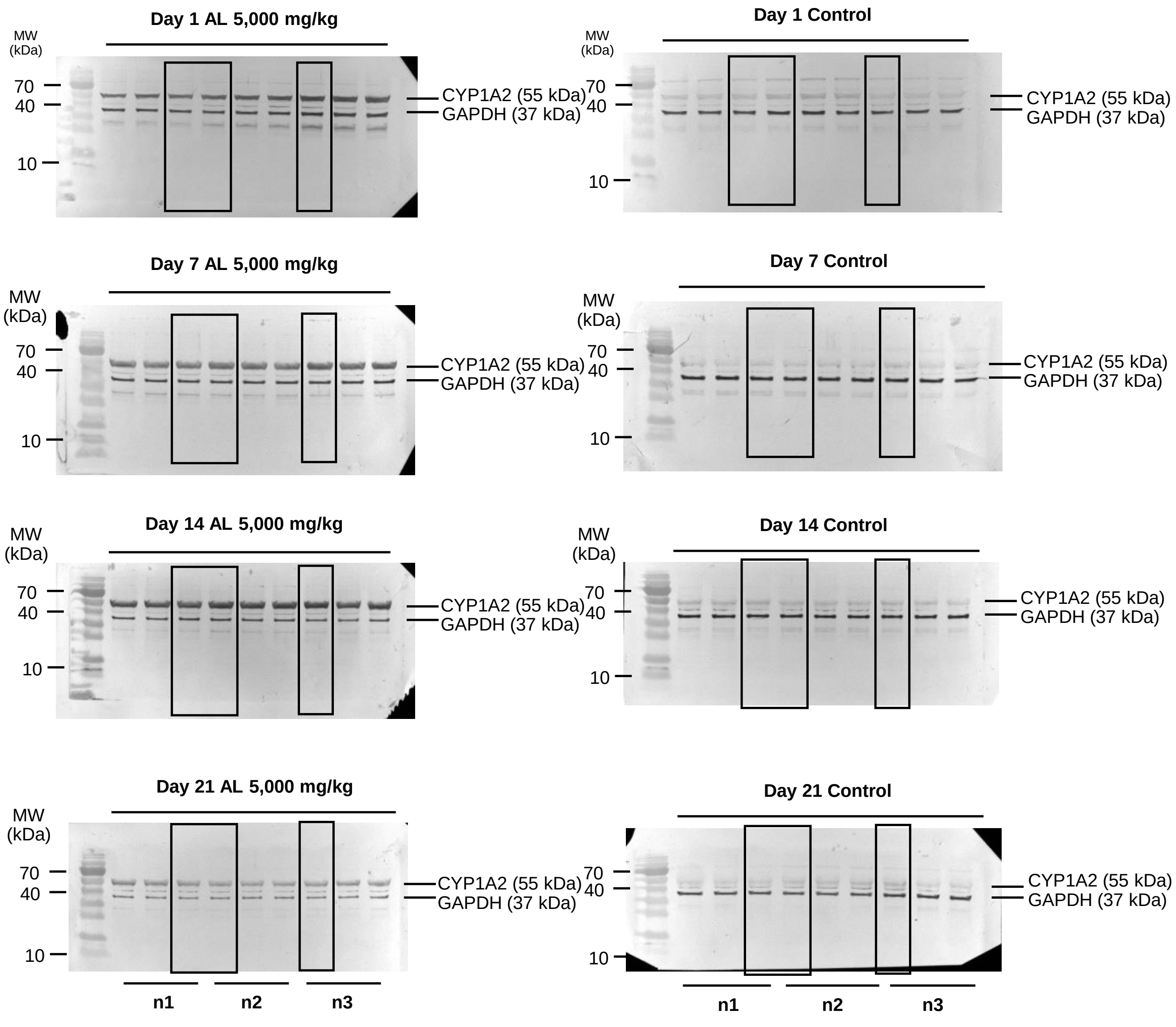

Fig 2C Western blot analysis of CYP3A1 in the livers of male and female WT rats treated with 1,000 (low dose), 3,000 (medium dose), 5,000 (high dose) mg/kg BW AL for 12 months. (Lanes in the boxes were cropped and presented in Fig 2C).

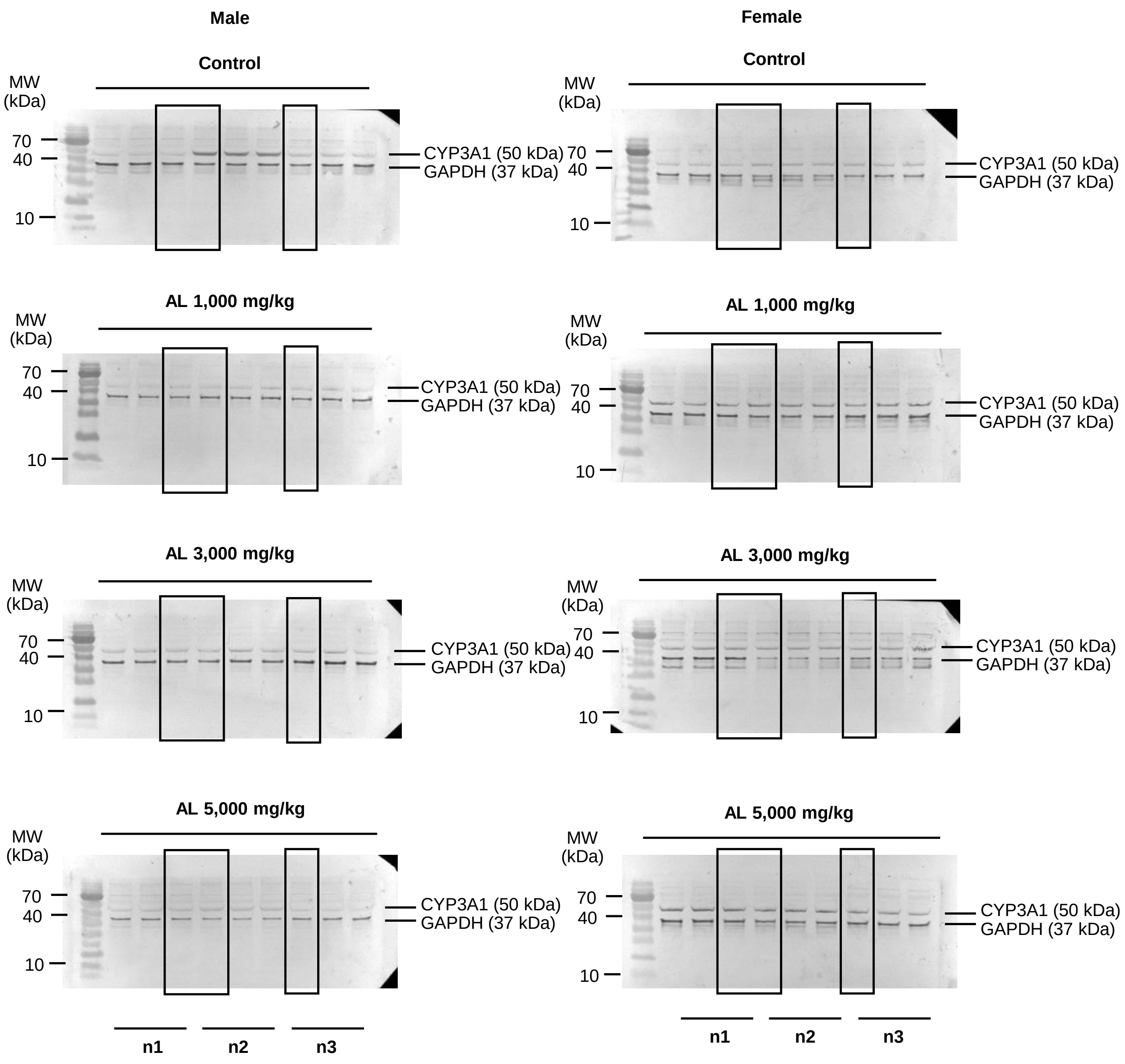

Fig 2C Western blot analysis of CYP1A2 in the livers of male SD rats treated with 5,000 mg/kg BW of placebo or AL for 1,7, 14, and 21 days. (Lanes in the boxes were cropped and presented in Fig 2C).

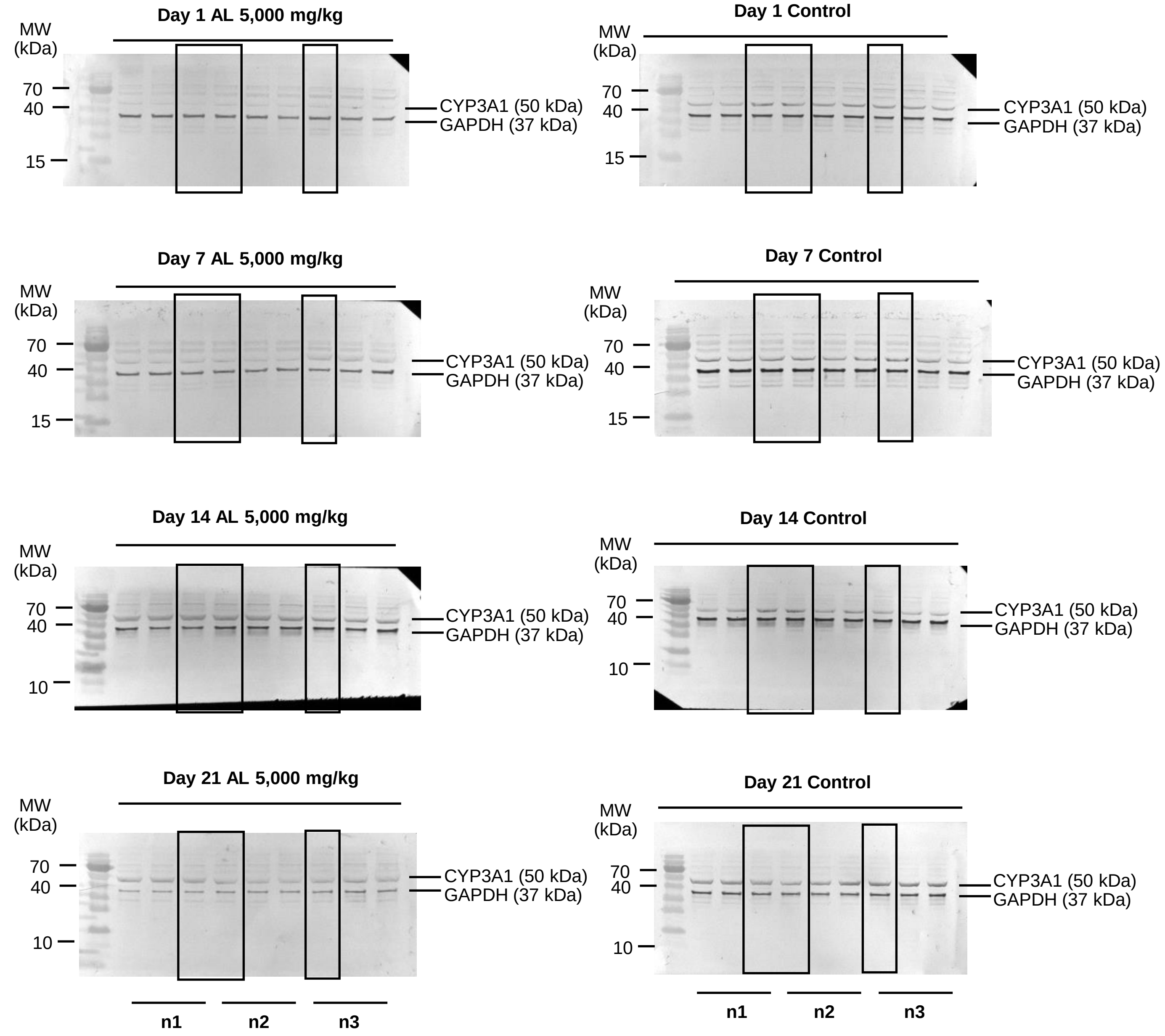

Supplement: S1 Raw images — (PDF) [file pone.0277614.s007.pdf]
